# Advancing Coronary Risk Assessment Through Combined Radiomic, Mechanical, and Hemodynamic Analysis

**DOI:** 10.1007/s10439-026-03994-1

**Published:** 2026-02-23

**Authors:** Anna Corti, Marco Stefanati, Vittorio Lissoni, Matteo Leccardi, Francesco Bruno, Alessandro Depaoli, Pietro Cerveri, Francesco Migliavacca, Valentina D. A. Corino, José F. Rodriguez Matas, Luca Mainardi, Gabriele Dubini

**Affiliations:** 1 Department of Electronics, Information and Bioengineering, Politecnico di Milano, Via Ponzio 34/5, 20133 Milan, Italy; 2 Laboratory of Biological Structure Mechanics (LaBS), Department of Chemistry, Materials and Chemical Engineering “Giulio Natta”, Politecnico di Milano, Milan, Italy; 3 Division of Cardiology, Department of Medical Sciences, “Città della Salute e della Scienza di Torino” Hospital, University of Turin, Turin, Italy; 4 Radiology Unit, Department of Surgical Sciences, “Città della Salute e della Scienza di Torino” Hospital, University of Turin, Turin, Italy; 5 Department of Industrial and Information Engineering, University of Pavia, Pavia, Italy; 6 Cardiotech Lab, Centro Cardiologico Monzino IRCCS, Milan, Italy

**Keywords:** Atherosclerotic plaque, Major adverse cardiac events, Radiomics, Finite element method, Computational fluid dynamics, Machine learning

## Abstract

**Purpose:**

Detecting vulnerable coronary plaques through coronary computed tomography angiography (CCTA) is a crucial, yet challenging task. To date, most of the proposed vulnerability markers have been studied in isolation. This study introduces the first integrated analysis combining radiomic, mechanical, and hemodynamic factors to explore their synergistic contribution to plaque vulnerability.

**Methods:**

The study analyzed 161 plaques in 46 coronary arteries from 39 patients, with 7 arteries (28 plaques) from 7 individuals, labeled as vulnerable from intravascular imaging. First, CCTA radiomic features were extracted. Second, mechanical markers were computed through finite element simulations conducted with varying material characteristics, accounting for the arterial wall mechanical properties’ uncertainties. Third, hemodynamic markers were derived from transient computational fluid dynamics simulations. Finally, a machine learning pipeline was developed to classify coronary arteries and patients based on radiomic, mechanical, and hemodynamic features, both individually and in combination.

**Results:**

Radiomics achieved the highest sensitivity (1.00), with all vulnerable patients identified, but lower specificity (0.69). Differently, mechanics and hemodynamics achieved higher specificities (0.94 and 0.97, respectively) but lower sensitivities (both 0.86). By integrating at least two out of the three models, the predictive performance improved, up to sensitivity = 1.00 and specificity = 0.97, with only one misclassified case.

**Conclusion:**

Although based on only 39 patients, the results highlight the power of a multi-level integrative approach for coronary plaque assessment. The study revealed that (i) hemodynamics outperformed mechanics and radiomics; (ii) while radiomics maximized sensitivity, mechanics and hemodynamics prioritized specificity, and (iii) integrating at least two variable types added value.

**Supplementary Information:**

The online version contains supplementary material available at 10.1007/s10439-026-03994-1.

## Introduction

Coronary artery disease (CAD) is the leading cause of death worldwide, responsible for 1087 over 1,000,000 deaths in 2021 [1]. The etiology is atherosclerosis, a chronic inflammatory-driven vascular disorder, characterized by the formation of fibro-fatty deposits within the arterial wall, known as atherosclerotic plaques [2]. While some plaques remain stable, growing and determining progressive lumen area reduction, the so-called “vulnerable plaques” are more prone to rupture phenomena causing major adverse cardiac events (MACE), such as myocardial infarction or death, regardless of the stenosis degree [3–6]. Thin-cap fibroatheromas (TCFA), presenting a large necrotic core surrounded by a thin fibrous cap (< 65 nm), have been identified as precursors of culprit plaques [7]. Additionally, large lipid core, spotty calcification, positive remodeling, intraplaque hemorrhage, macrophage infiltration, and neovascularization represent other hallmarks of vulnerable plaques [8].

Coronary computed tomography angiography (CCTA) has rapidly emerged as a powerful non-invasive imaging modality for CAD diagnosis and the vulnerable coronary plaque assessment [4, 9]. Particularly, the presence of at least two CCTA-based features among positive remodeling, low attenuation, spotty calcification, and ‘napkin-ring’ sign suggests a condition of high-risk plaque [10, 11]. Additionally, several CCTA-based risk scores have been proposed for coronary plaque stratification, based on stenosis degree, plaque composition, burden, or location [12], including the CAD-RADS [13], the CT-adapted Leaman score [14, 15], or the Leiden CCTA score [16].

Due to the complexity of the atherosclerotic scenario, characterized by the interrelation of biological, morphological and mechanical factors, various vulnerability markers have been explored [17–22]. Among these, biomechanical features, as structural and hemodynamic markers, have been largely investigated [23–31], identifying high plaque structural stress as associated with vulnerable plaques and risk of MACE [22–28], low wall shear stress (WSS) as a driver of plaque progression, and high WSS as related to plaque vulnerability [32–36]. However, their predictive role has been under-investigated: while statistical associations were found, machine learning (ML) models based on structural mechanics or hemodynamic features to predict coronary plaque vulnerability are still rare in the literature [17]. The emerging field of radiomics has also recently shown promise in identifying vulnerable coronary plaques through the extraction and mining of quantitative features capturing information about plaque components, tissue composition, and morphological characteristics from CCTA images [37, 38].

To date, research has primarily investigated individual markers of plaque vulnerability in isolation, although the complex vulnerability condition is more likely driven by the synergistic interaction of multiple contributing factors. In our previous investigation, we demonstrated that the combination of radiomics and structural mechanics improved the identification of high-risk arteries and patients, over single factors alone [39]. However, the study neglected the role of hemodynamics and considered average mechanical properties of the plaque components, being a major limitation [39]. This study marks a significant advancement by introducing, for the first time, a combined radiomic–mechanic–hemodynamic predictor of coronary vulnerability. It addresses gaps in previous research by: (i) exploring the predictive performance of WSS-based descriptors, computed by computational fluid dynamics (CFD), either alone or in combination with radiomic and mechanical markers, and (ii) improving the mechanical-based model, through a data-augmentation approach that accounts for the uncertainty of mechanical properties.

## Materials and Methods

### Patient Dataset

A single-center, prospective study was performed in the years 2020–2021. Thirty-nine patients who underwent both CCTA and optical coherence tomography (OCT) at the Azienda Ospedaliero-Universitaria “Città della Salute e della Scienza” of Torino, Italy, were included. The protocols were approved by the Ethical Committee (Prot. no. 0068508), and data acquisition followed the General Data Protection Regulation of the EU. All patients signed the informed consent.

Each patient presented plaques in at least one coronary artery (left ascending, left circumflex, and/or right coronary), leading to 46 coronary arteries with 161 plaques. In case of at least one plaque identified as vulnerable by OCT, the specific coronary artery was labeled as “vulnerable,” and the patient as “high risk”: 7 vulnerable coronary arteries with 28 plaques were identified, belonging to 7 high-risk patients [39].

CCTA was performed using a Revolution CT (GE Healthcare, Milwaukee, WI, USA), and OCT with a CT system (LightLab Imaging Inc/St Jude Medical, Westford, MA, USA); further details are provided in the Supplementary Materials. OCT images were analyzed by an expert operator at the time of the procedure, and post hoc offline by an independent investigator blinded to the outcome. The culprit lesion was identified through angiography, electrocardiographic ST-segment alterations, and/or regional wall motion abnormalities on echocardiography. Plaque rupture was defined as the presence of fibrous cap discontinuity leading to exposure of the necrotic core to the lumen, or disruption over a calcified plaque. TCFA at the rupture site was recorded. Fibrocalcific, fibrotic plaque, lipid component, or macrophage infiltration was defined according to IWG-IVOCT Consensus standards [40] (Table 1).

**Table 1:** Patient clinical characteristics

Patient characteristics	Set (39)
Age	64.28 (SD 10.6)
Sex	
Male	28 (72%)
Female	11 (28%)
Diabetes	7 (17.9%)
Dyslipidemia	27 (69.2%)
Smoking status	
Current/former	20 (51.3%)
Never	19 (48.7%)
Family history of CAD	17 (43.6%)
Hypertension	32 (82%)
Obesity	11 (28.2%)
AF	6 (15.4%)
PAD	4 (10.3%)
PCI	29 (74.4%)
Adverse events	(23%)
CV death	2
Other death	2
MI	1
Urgent revascularization	3
Stroke	1
Patient risk	
High	7 (18 %)
Low	32 (82 %)

### Image Segmentation

CCTA images were semi-automatedly segmented through the plaque analysis software QAngioCT Research Edition (Medis Medical Imaging software, Leiden, The Netherlands). Specifically, the coronary lumen, external surface, and the surfaces delimiting the arterial wall components (medial/normal wall, fibrous, fibrous fatty, lipid, and calcific tissues) were identified and exported as stereolithographic files (by using the 3D Workbench package) to be used for subsequent radiomic, structural mechanics, and hemodynamic analyses.

### Radiomic Analysis

For each plaque, the region of interest for the radiomic analysis consisted of the arterial wall segment delimited by the proximal and distal plaque locations as identified by expert radiologists. Before feature extraction, CCTA images were preprocessed by applying the following: (i) 3D Gaussian filter with a 3 × 3 × 3 voxel kernel and *σ* = 0.5; (ii) 25-width bin histogram discretization; and (iii) 2 mm isotropic voxel size resampling, with B-spline interpolation [41]. Pyradiomics 2.2.0 (https://github.com/Radiomics/pyradiomics, run on Python) [42] was used to extract 107 radiomic features: 14 shape and size, 18 first-order statistics, and 75 textural features (24 gray level co-occurrence matrix, 16 gray level run length matrix, 14 gray level dependence matrix, 5 neighboring gray tone difference matrix, and 16 gray level size zone matrix features) [43].

### Structural Mechanics Analysis

**Fig. 1:**
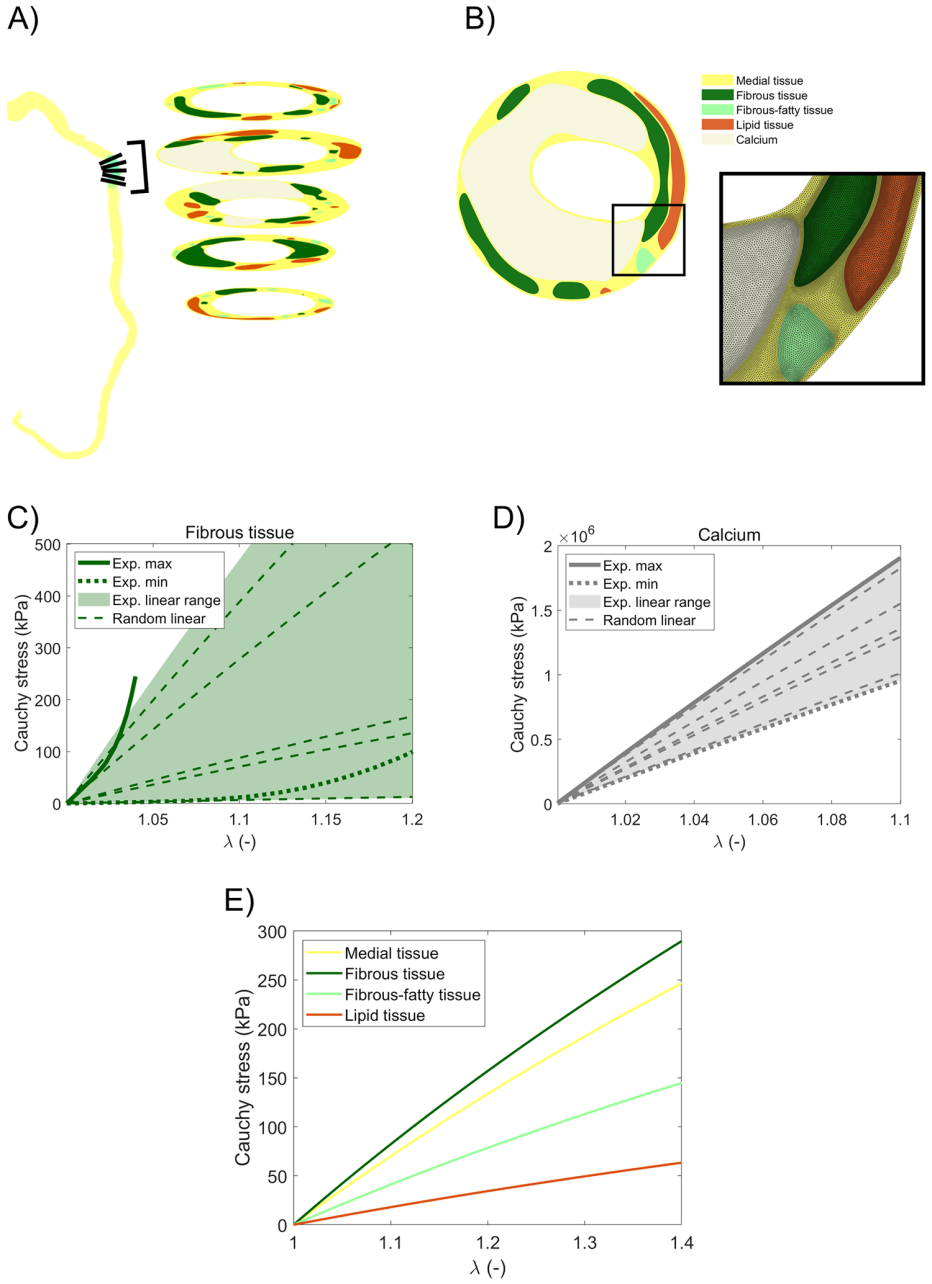
**A** Five evenly distributed coronary cross-sections of a representative plaque. **B** Representation of an exemplifying coronary cross-section with five arterial wall components: medial tissue (yellow), fibrous tissue (dark green), fibrous fatty tissue (light green), lipid tissue (red), and calcifications (white). Magnification of a portion of the arterial wall with representation of the triangular mesh. **C** Stretch–stress curves of the fibrous tissue, with details on the experimental linear range (colored area), obtained from the linearization of the hyperelastic literature-derived maximum and minimum curves (Exp. max and Exp. min). The dashed curves represent random linear curves. **D** Stretch–stress curves of the calcification, with details on the experimental linear range (colored area), obtained from the linearization of the hyperelastic literature-derived maximum and minimum curves (Exp. max and Exp. min). The dashed curves represent random linear curves. **E** Stretch–stress linear averaged curves of the medial, fibrous, fibrous fatty, and lipid tissues. The stretch *λ* is equal to *ε* + 1

The structural mechanics analysis relied on our previous studies [22, 39]. Briefly, five evenly distributed sections were considered for each plaque and modeled as composed of medial (normal wall), fibrous, fibrous fatty, lipid (necrotic core) tissues, and calcifications (Fig. 1). Each tissue component was modeled as linear isotropic and quasi-incompressible material (Poisson’s ratio *ν* = 0.475 for all materials, except for calcifications, *ν* = 0.3) [44]. To account for the variability and uncertainty of the mechanical properties and its impact on the vulnerability prediction, 100 values for the Young’s Modulus (E) were defined for each tissue component within the literature-derived range reported in Table 2 (Fig. 1) [44, 45]. Subsequently, 100 different random combinations of the five tissue components were defined. An in-house method, developed in MATLAB (The MathWorks Inc, Natick, MA, USA), was used to generate the triangular mesh with an average element size of 0.0131 mm (maximum of 0.0502 mm and minimum of 0.00125 mm) for finite element analysis (FEA) [22]. Patient-specific diastolic and systolic pressures were imposed at the luminal surface, and Robin boundary condition was prescribed at the external surface [22, 46].

**Table 2:** Tissue young modulus (E)

Tissue	Min E (kPa)	Max E (kPa)	References
Medial tissue	8.9 × 10^1^	3.6 × 10^3^	[45]
Fibrous tissue	5.8 × 10^1^	4.9 × 10^3^	[45]
Fibrous-fatty tissue	2.9 × 10^1^	2.5 × 10^3^	[45]
Lipid core	1.3 × 10^1^	9.5 × 10^2^	[45]
Calcifications	1.0 × 10^7^	2.0 × 10^7^	[44]

For each cross-section, 100 FEA simulations with variable material combinations were performed, leading to the computation of 100 values of maximum dynamic von Mises stress ($\mathrm{max}\_{pMISES}^{dyn}$) and maximum dynamic equivalent strain (max_${\varepsilon }_{eqv}^{dyn})$, computed as the difference between the values obtained at the systolic and diastolic pressures (${P}_{sys}$ and ${P}_{dia}$), with $MISES$ and ${\varepsilon }_{eqv}$ defined in Table 3. Then, for each plaque, 100 instances were obtained by averaging $\mathrm{max}\_{pMISES}^{dyn}$ and max_${\varepsilon }_{eqv}^{dyn}$ over the five cross-sections with the same material combination.

**Table 3:** Von Mises Stress (MISES) and equivalent strain (${\varepsilon }_{eqv})$ computation

Variable	Formulation
$MISES$	$\frac{1}{\sqrt{2}}\sqrt{{\left({\sigma }_{xx}-{\sigma }_{yy}\right)}^{2}+{\left({\sigma }_{yy}-{\sigma }_{zz}\right)}^{2}+{\left({\sigma }_{zz}-{\sigma }_{xx}\right)}^{2}+6\left({\sigma }_{xy}^{2}+{\sigma }_{yz}^{2}+{\sigma }_{zx}^{2}\right)}$
${\varepsilon }_{eqv}$	$\frac{1}{\sqrt{2}\left(1+\nu \right)}\sqrt{{\left({\varepsilon }_{xx}-{\varepsilon }_{yy}\right)}^{2}+{\left({\varepsilon }_{yy}-{\varepsilon }_{zz}\right)}^{2}+{\left({\varepsilon }_{zz}-{\varepsilon }_{xx}\right)}^{2}+6\left({\varepsilon }_{xy}^{2}+{\varepsilon }_{yz}^{2}+{\varepsilon }_{zx}^{2}\right)}$

Besides $\mathrm{max}\_{pMISES}^{dyn}$ and $\mathrm{max}$_${\varepsilon }_{eqv}^{dyn}$, the patient’s ${P}_{sys}$, ${P}_{dia} ,$ and dynamic pressures (${P}_{dyn}={P}_{sys}-{P}_{dia}$), were considered. The final mechanical feature set consisted in 161 plaques × 100 instances × 5 features. Further details on the definition of the cross-sections and FEA simulations are provided in the Supplementary Material.

### Computational Fluid Dynamics Analysis

**Fig. 2:**
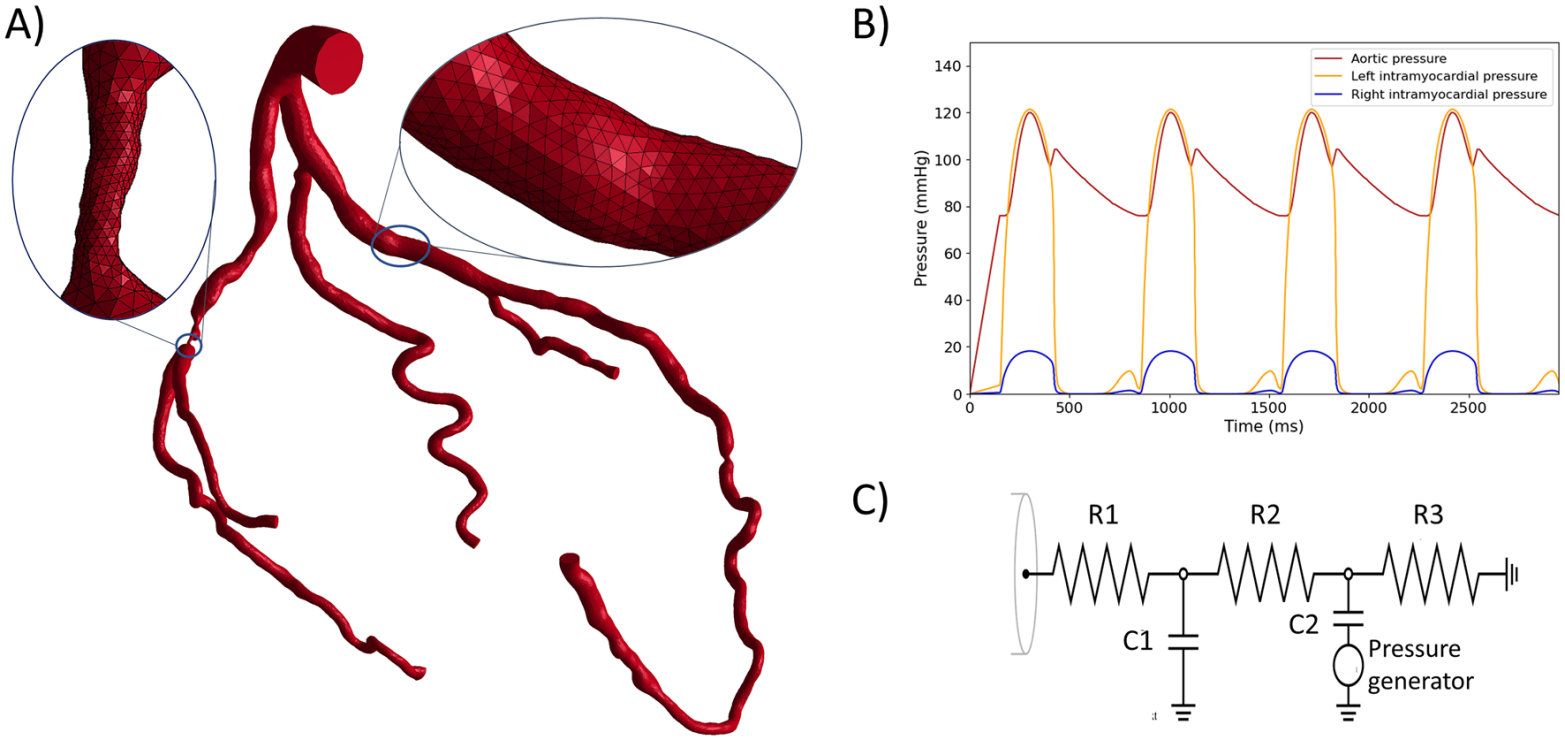
Example of the computational fluid dynamic model of one patient. **A** Coronary artery tree with details of the tetrahedral mesh and mesh refinement in correspondence of the stenosis. **B** Aortic, left intramyocardial, and right intramyocardial pressures. **C** Five-element lumper parameter model

Each coronary artery tree model was meshed using a triangular surface mesh (average element size: 0.45 mm) with local refinements near the stenosis; a tetrahedral volumetric mesh was then generated in LS-DYNA with a growth factor of 1.0 and a three-elements boundary layer (Fig. 2). CFD simulations were performed using the finite element solver of ICFD module in LS-DYNA 971 14.0 (ANSYS, Canonsburg, PA, USA). Flow was modeled as laminar (Reynolds < 2000). Blood was modeled as a Newtonian fluid with a viscosity of 3.5 × 10^−3^ Pa·s and a density of 1063.5 kg/m^3^. The physiological aortic pressure curve [47] was scaled with patient-specific diastolic and systolic pressure and heart rate and imposed as an inlet boundary condition, along with a no-slip condition on the vessel walls (Fig. 2B). A five-element lumped parameter model [48–50] was prescribed at the outlets to simulate the downstream microvasculature, based on coronary blood flow data from the literature (Fig. 2C) [51]. This model consists of three resistances (R1, R2, R3), two compliances (C1, C2), and a pressure generator, simulating the intramyocardial pressure to modulate blood flow [52]. For left and right coronaries, the physiological left [47] or right intraventricular pressure [52] was scaled based on the patient-specific systolic pressure. The total resistance *R*_tot_ was adjusted to match the patient-specific average aortic pressure and average coronary flow rate from the literature [51]. Blood flow was distributed among the outlets according to Murray’s Law [53, 54]. Consequently, *R*_tot_ was partitioned among the different outlets (Table 4) following the same methodology presented by Taylor et al. [55]. In a previous study of ours [56], we found that the *C*_*i*_ relationship reported in Table 4 provides the optimal outlet total compliance, ensuring that the simulated flow curves closely match the theoretical ones [51]. *R*_*i*_ and *C*_*i*_ at each outlet were then split into R1, R2, R3, and C1 and C2, respectively, according to specific percentage values from the literature [50]. Details on the mesh sensitivity analysis, solver, convergence criteria, and computational requirements for simulations are provided in the Supplementary Material.

**Table 4:** Five-element lumped parameter model components

Component	Formulation
Total resistance of the coronary artery	$Rtot=\frac{Paverage(patient-specific)}{Qaverage(literature)}$
Resistance of the i-th outlet section	${R}_{i}= {\left(\frac{{{Diameter}_{i}}^{3}}{\sum {Diameter}^{3}}\right)}^{-1}Rtot$
Compliance of the i-th outlet section	$Ci=\frac{100}{Ri\cdot dt}$
Resistance components in the i-th outlet section	$R1=0.32\cdot Rtot$, $R2=0.52\cdot Rtot,$ $R3=0.16\cdot Rtot$
Compliance components in the i-th outlet section	$C1=0.11\cdot Ctot$, $C2=0.89\cdot Ctot$

Three cardiac cycles were simulated for each coronary, and results were extracted from the last one. WSS-based descriptors (Table 5), namely the time-averaged WSS (TAWSS), the oscillatory shear index (OSI), the relative residence time (RRT), and the transverse WSS (TransWSS), were computed in Paraview. To identify thresholds for “disturbed” hemodynamics, data from all the cases were pooled, and the 33rd and 66th percentile values of each descriptor were computed. Plaque segments were isolated, and for each segment, the minimum, maximum, median, mean, standard deviation, and surface area (both in mm and in percentage) exposed to values < 33rd or > 66th percentiles were computed for each hemodynamic descriptor, resulting in 36 hemodynamic features. More details of the implementation, boundary condition values, and results extracted are reported in the Supplementary Material, with two examples of different coronaries.

**Table 5:** Wall shear stress (WSS) descriptors

TAWSS	$TAWSS= {\int }_{0}^{T}\left|WSS\left(s,t\right)\right|\cdot dt$
OSI	$OSI=0.5\cdot \left[1-\left(\frac{\left|{\int }_{0}^{T}WSS\left(s,t\right)\cdot dt\right|}{{\int }_{0}^{T}\left|WSS\left(s,t\right)\right|\cdot dt}\right)\right]$
RRT	$RRT=\frac{1}{\left(1-2\cdot OSI\right)\cdot TAWSS}=\frac{T}{\left|{\int }_{0}^{T}WSS\left(s,t\right)\cdot dt\right|}$
TransWSS	$transWSS=\frac{1}{T}\cdot {\int }_{0}^{T}\left|WSS\left(n x \frac{{\int }_{0}^{T}WSS\cdot dt}{\left|{\int }_{0}^{T}WSS\cdot dt\right|} \right)\right|\cdot dt$

*WSS*: wall shear stress, *TAWSS*: time-averaged WSS, *OSI*: oscillatory shear index, *RRT*: relative residence time, *TransWSS*: transverse WSS

### Machine Learning Models’ Development

**Fig. 3:**
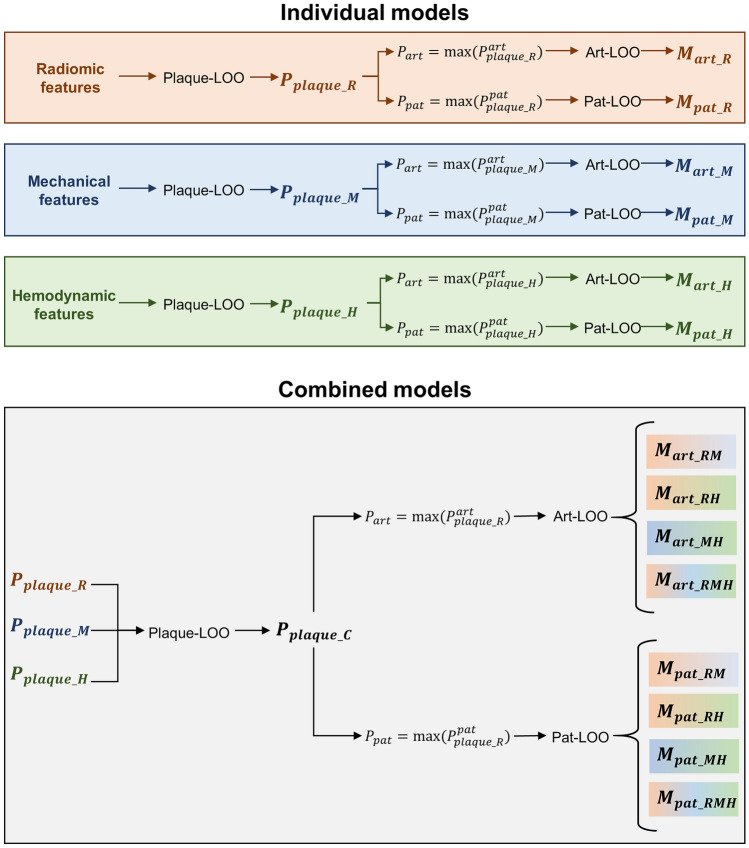
Machine learning pipeline for the development of the radiomic, mechanical, hemodynamic, and combined predictive models. Plaque-LOO: leave-one-out cross-validation at the plaque level; Art-LOO: leave-one-out cross-validation at the artery level; Pat-LOO: leave-one-out cross-validation at the patient level; P_plaque_R_: radiomic-based plaque probability to belong to a vulnerable coronary artery; P_plaque_M_: mechanical-based plaque probability to belong to a vulnerable coronary artery; P_plaque_H_: hemodynamic-based plaque probability to belong to a vulnerable coronary artery; P_plaque_C_: combined plaque probability to belong to a vulnerable coronary artery; M_art_R_: radiomic-based artery stratification model; M_art_M_: mechanical-based artery stratification model; M_art_H_ hemodynamic-based artery stratification model; M_art_C_: combined artery stratification model; M_pat_R_: radiomic-based patient stratification model; M_pat_M_: mechanical-based patient stratification model; M_pat_H_: hemodynamic-based patient stratification model; M_pat_C_: combined patient stratification model

The two-step ML pipeline previously proposed [39] was adapted to stratify coronary arteries (vulnerable vs. non-vulnerable) and patients (high risk vs. low risk), by considering either individual radiomic, mechanical, hemodynamic, or combined features (radiomic–mechanical, radiomic–hemodynamic, mechanical–hemodynamic, radiomic–mechanical–hemodynamic), as shown in Fig. 3.

For the individual models, the first step consisted of a leave-one-out cross-validation at the plaque level (plaque-LOO) for the computation of the predicted plaque vulnerability probability, and the second step consisted of a leave-one-out cross-validation at the artery (art-LOO) or patient level (pat-LOO) for the final artery/patient stratification. The plaque-LOO step was applied to the 161 plaques for the radiomic and hemodynamic models and to the 16,100 plaque instances for the mechanical model. For the latter, each iteration used the 100 instances of one plaque for testing and the 16,000 instances from the remaining plaques for training. At each iteration of the plaque-LOO step, on the training set, (i) features were Z-score normalized, and the used mean and standard deviation were applied for normalization in the test set; (ii) dimensionality reduction was obtained by applying feature selection; (iii) data balancing was applied through a random undersampling of the majority class (to 60 samples for radiomic/hemodynamic cases and 4000 samples for the mechanical case), followed by the oversampling of the minority class using the synthetic minority oversampling technique [57]; and (iv) ML classifiers were trained. As regards the feature selection, the significantly different features between the plaques in the vulnerable and non-vulnerable arteries were identified using the Mann–Whitney *U* test (*p* < 0.05). In case of absence or numerous significant features, the first 4 features in ascending *p*-value order were considered, as in [39]. Regarding ML classifiers, K-Nearest Neighbor (KNN), Decision Tree (DT), and AdaBoost (ADA) algorithms were trained for the radiomic, mechanical, and hemodynamic models through a fivefold cross-validation on the training set [39]. The trained models were then applied to the test plaque (or plaque instances) for the computation of the vulnerability probabilities: *P*_plaque_R_, *P*_plaque_M_, and *P*_plaque_H_ for the radiomic, mechanical, and hemodynamic models, respectively. For the mechanical model, *P*_plaque_M_ was computed as the average of the 100 predicted probabilities for the plaque instances.

The art/pat-LOO step (Fig. 3) consisted of the artery/patient stratification by considering the plaque with the highest probability *P*_plaque_ and estimating the optimal probability threshold for the binary classification, based on the maximum Youden’s *J* value (difference between the true positive and false positive rates). The threshold was estimated at each iteration of the training set (consisting of *N*_art_ − 1 or *N*_pat_ − 1, in case of the artery/patient stratification task) and then applied to classify the artery/patient in the test set, leading to the final radiomic, mechanical, and hemodynamic models for the arteries (*M*_art_R_, *M*_art_M_, and *M*_art_H_) and patients (*M*_pat_R_, *M*_pat_M_, and *M*_pat_H_).

For the combined models, the same plaque-LOO and art/pat-LOO steps were applied but considering the predicted probabilities *P*_plaque_R_, *P*_plaque_M_, and *P*_plaque_H_ as features, without performing feature selection (Fig. 3). Thus, *P*_plaque_R_ and *P*_plaque_M_ were used to train the radiomic–mechanical model, *P*_plaque_R_ and *P*_plaque_H_ for the radiomic–hemodynamic model, *P*_plaque_M_ and *P*_plaque_H_ for the mechanical–hemodynamic model, and all three for the radiomic–mechanical–hemodynamic model. The ML classifiers used for the individual models were considered for the combined models, and the best performing algorithm was selected.

The predictive performance of the models was evaluated through the balanced accuracy, sensitivity, specificity, and Area Under the Curve of the Receiver Operating Characteristic curve (AUC-ROC). McNemar’s test was used to compare the confusion matrix of the developed models [58].

## Results

### Radiomic, Mechanical, and Hemodynamic Features

**Fig. 4:**
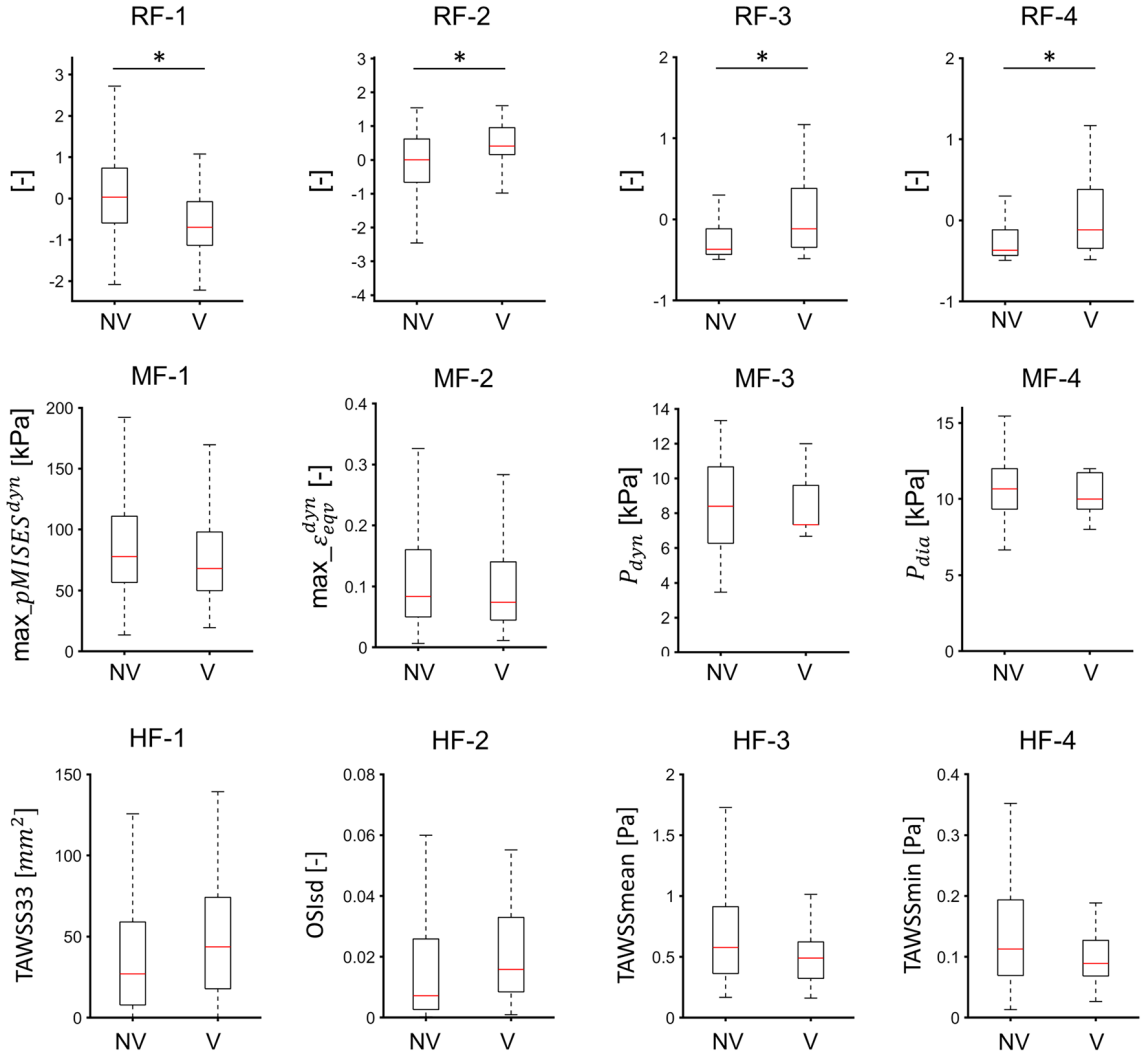
Boxplots of the four most occurring radiomic features (RF), the four selected mechanical features (MF) and the four most occurring hemodynamic features (HF) for the plaques in non-vulnerable (NV) and vulnerable (V) arteries. RF-1: *original_glszm_GrayLevelNonUniformityNormalized*; RF-2: *original_glcm_Correlation*; RF-3: *original_firstorder_TotalEnergy*; RF-4: *original_firstorder_Energy*; MF-1: $\mathrm{max}\_{pMISES}^{dyn}$; MF-2: max_${\varepsilon }_{eqv}^{dyn}$; MF-3: ${P}_{dyn}$; MF-4: ${P}_{dia}$; HF-1: TAWSS33 (area exposed to a time-averaged wall shear stress < 33rd percentile); HF-2: OSIsd (standard deviation of oscillatory shear index); HF-3: TAWSSmean (mean time-averaged wall shear stress); HF-4: TAWSSmin (minimum time-averaged wall shear stress). RFs have been z-score normalized. *p-value < 0.05

**Fig. 5:**
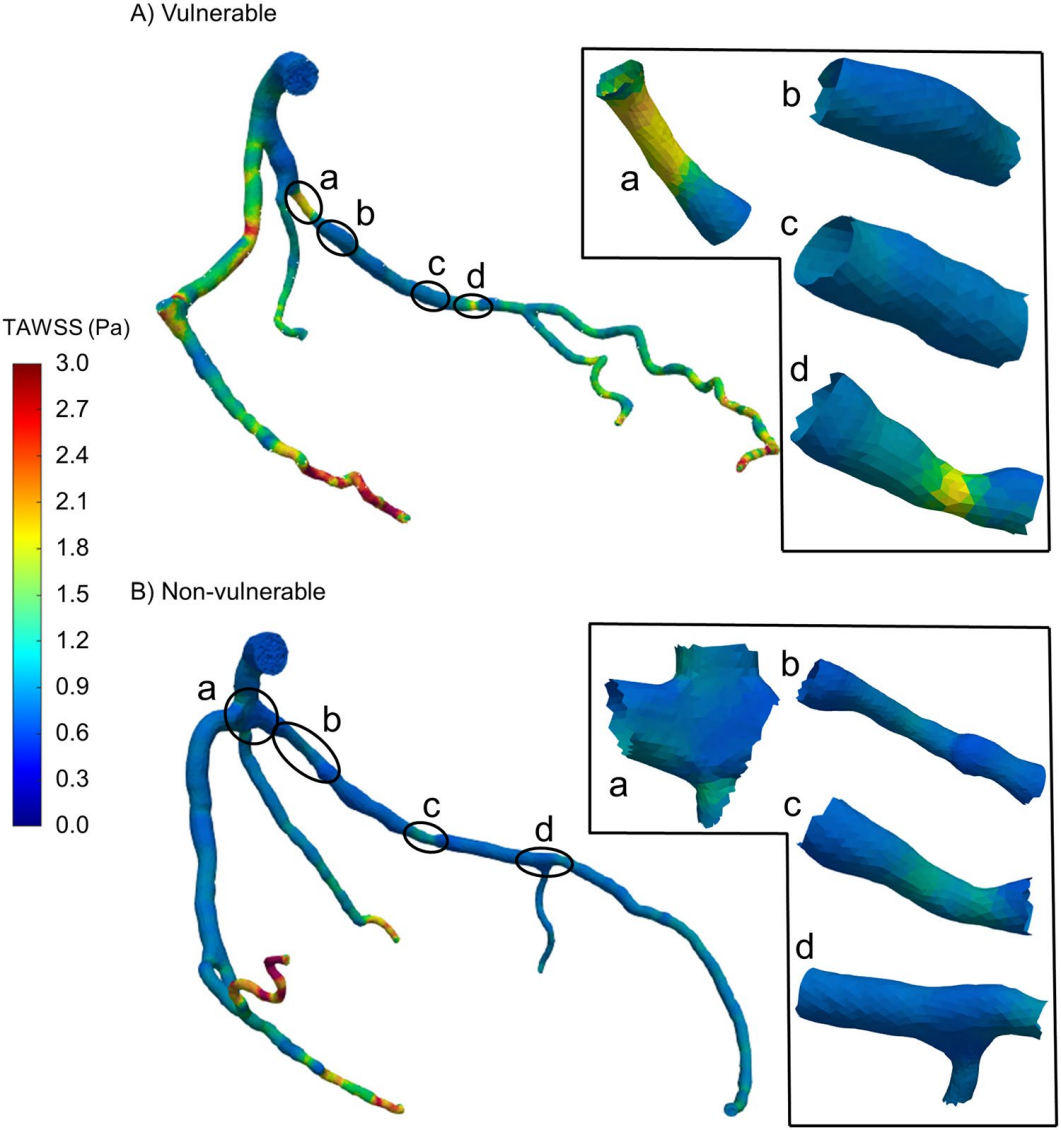
Time-averaged wall shear stress (TAWSS) contour in two explanatory coronary trees and corresponding plaques. **A** Vulnerable coronary artery; **B** Non-vulnerable coronary artery

At each iteration of the plaque-LOO step, four most significant radiomic, mechanical, and hemodynamic features were selected. Across all the iterations, ten different radiomic features were globally identified, four of which occurred in more than 150 iterations: the *glszm_GrayLevelNonUniformityNormalized*, a textural feature measuring the variability of gray-level intensity values in the image; the *glcm_Correlation*, a textural feature measuring the linear dependency of gray-level values to their respective voxels; and the *TotalEnergy* and *Energy*, both first-order features associated with the magnitude of voxel values in an image. All the five mechanical features were identified with ${P}_{dia}$, ${P}_{dyn}$, $\mathrm{max}\_{pMISES}^{dyn}$, and max_${\varepsilon }_{eqv}^{dyn}$ being the most occurring across the iterations. Finally, five hemodynamic features were identified: the area exposed to a TAWSS < 33rd percentile (TAWSS33); standard deviation of OSI (OSIsd); mean TAWSS (TAWSSmean); minimum TAWSS (TAWSSmin); and median TAWSS (TAWSSmedian). Of these, TAWSS33, OSIsd and TAWSSmean were selected across all the iterations, TAWSSmin in 154 over 161 iterations, while TAWSSmedian in only 7 over 161 iterations. Figure 4 shows the distribution of the four most occurring radiomic, mechanical, and hemodynamic features within the non-vulnerable and vulnerable groups. While all the radiomic features were significantly different between the groups (*p* < 0.05), the mechanical and hemodynamic features did not show statistically significant differences (*p* > 0.05). Figure 5 depicts the TAWSS contour in a vulnerable and non-vulnerable case, with plaques in the vulnerable artery presenting higher TAWSS than those in the non-vulnerable artery. Further details on the hemodynamic features of these exemplifying coronary arteries are provided in the Supplementary Tables S5–S8.

**Fig. 6:**
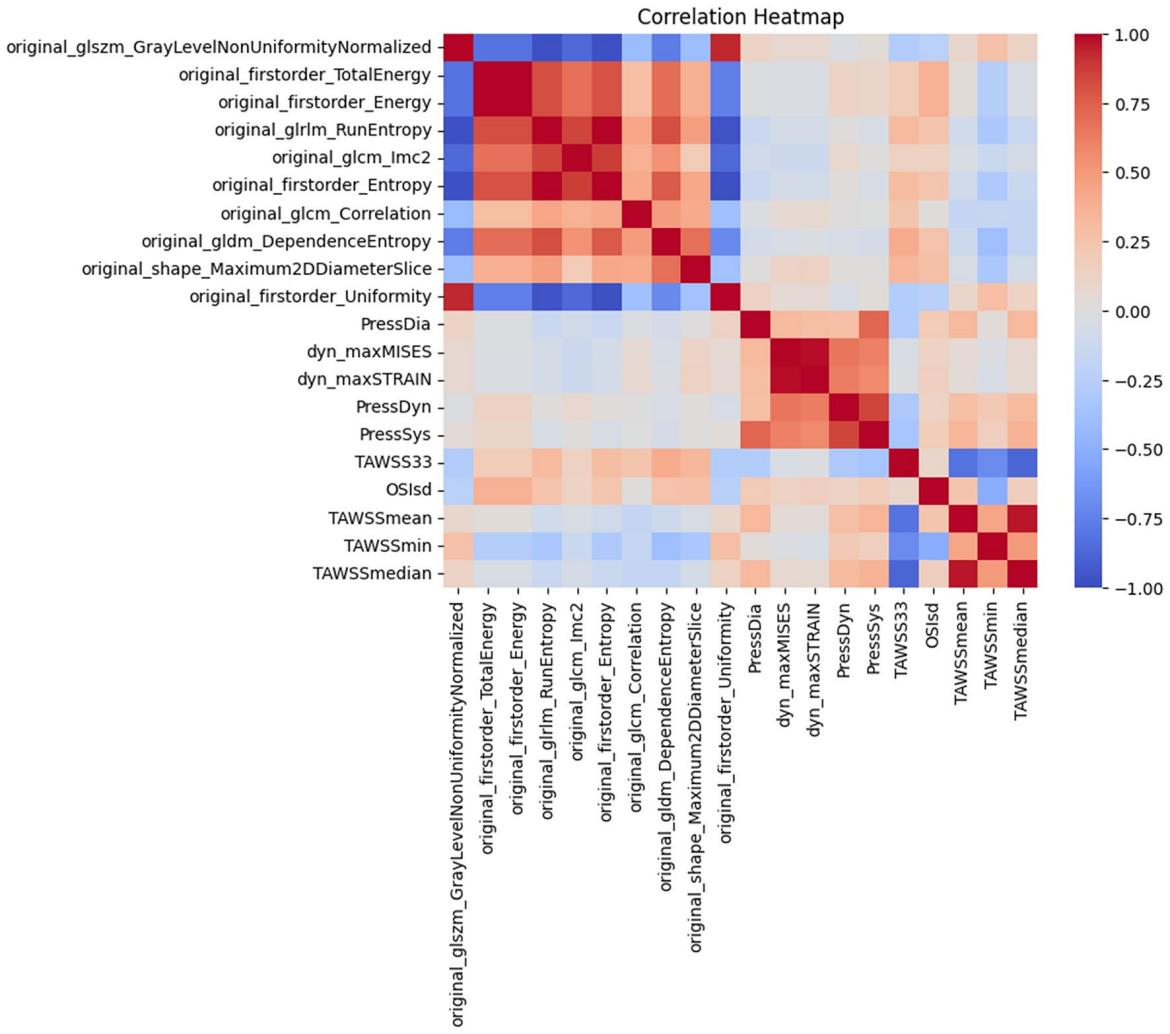
Spearman’s correlation coefficient among the selected radiomic, mechanical, and hemodynamic features

Figure 6 shows the correlation (Spearman’s coefficient) among the radiomic, mechanical, and hemodynamic features: while features within each domain were correlated, no correlations were identified among features of the three domains. High correlations (|*ρ*|>0.80) were found across all radiomic features, with the exception of *glcm_Correlation* and *shape_maximum2DDiameterSlice* which were scarcely correlated with all the other radiomic features. High correlations were also identified between $\mathrm{max}\_{pMISES}^{dyn}$ and max_${\varepsilon }_{eqv}^{dyn}$, and between ${P}_{sys}$ and ${P}_{dyn}$. Finally, as expected, the TAWSSmedian and TAWSSmean showed a strong correlation with each other and were also highly correlated with TAWSS33.

### Artery and Patient Vulnerability Prediction

**Fig. 7:**
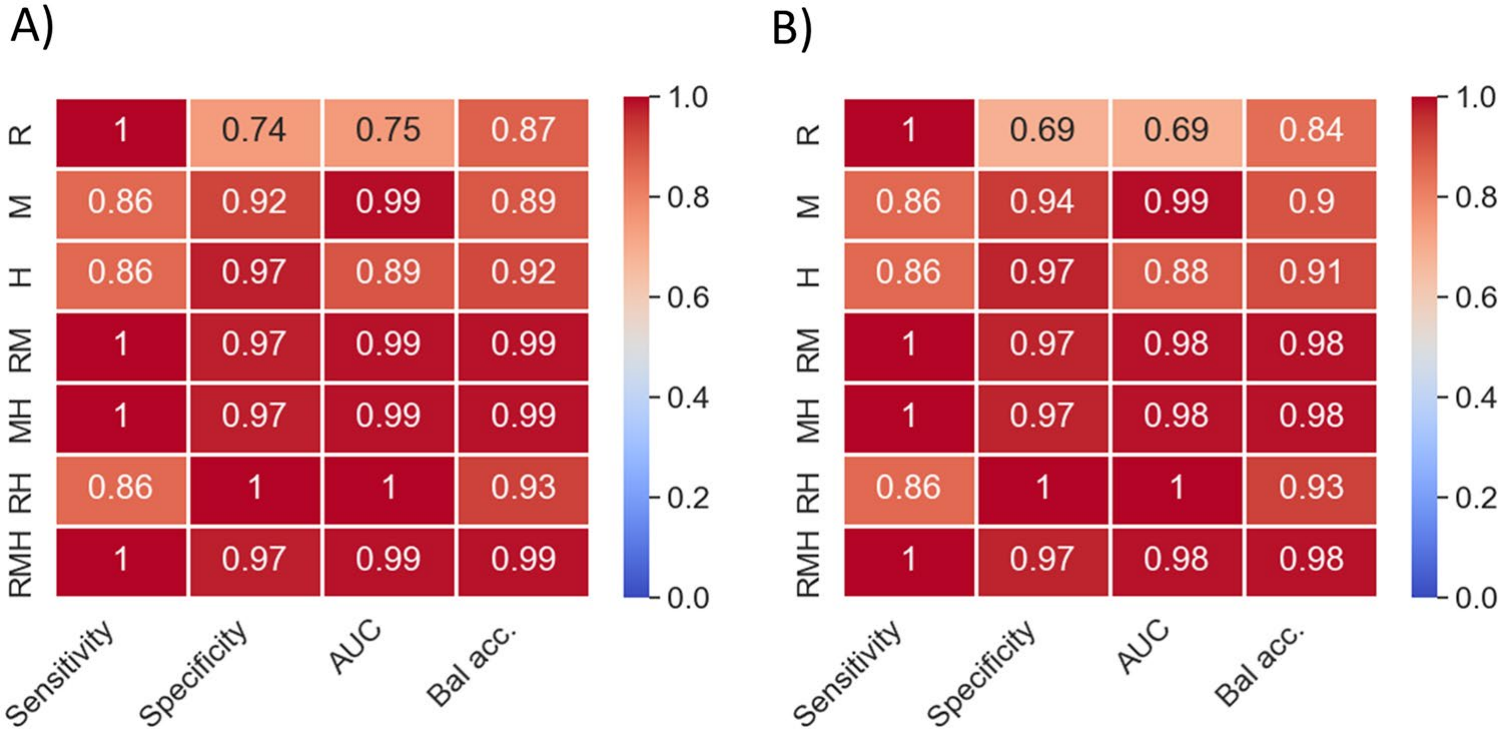
Performance metrics of the radiomic (R), mechanical (M), hemodynamic (H), radiomic–mechanical (RM), mechanical–hemodynamic (MH), radiomic–hemodynamic (RH), and radiomic–mechanical–hemodynamic (RMH) models for the artery stratification (**A**) and patient stratification (**B**) cases

**Fig. 8:**
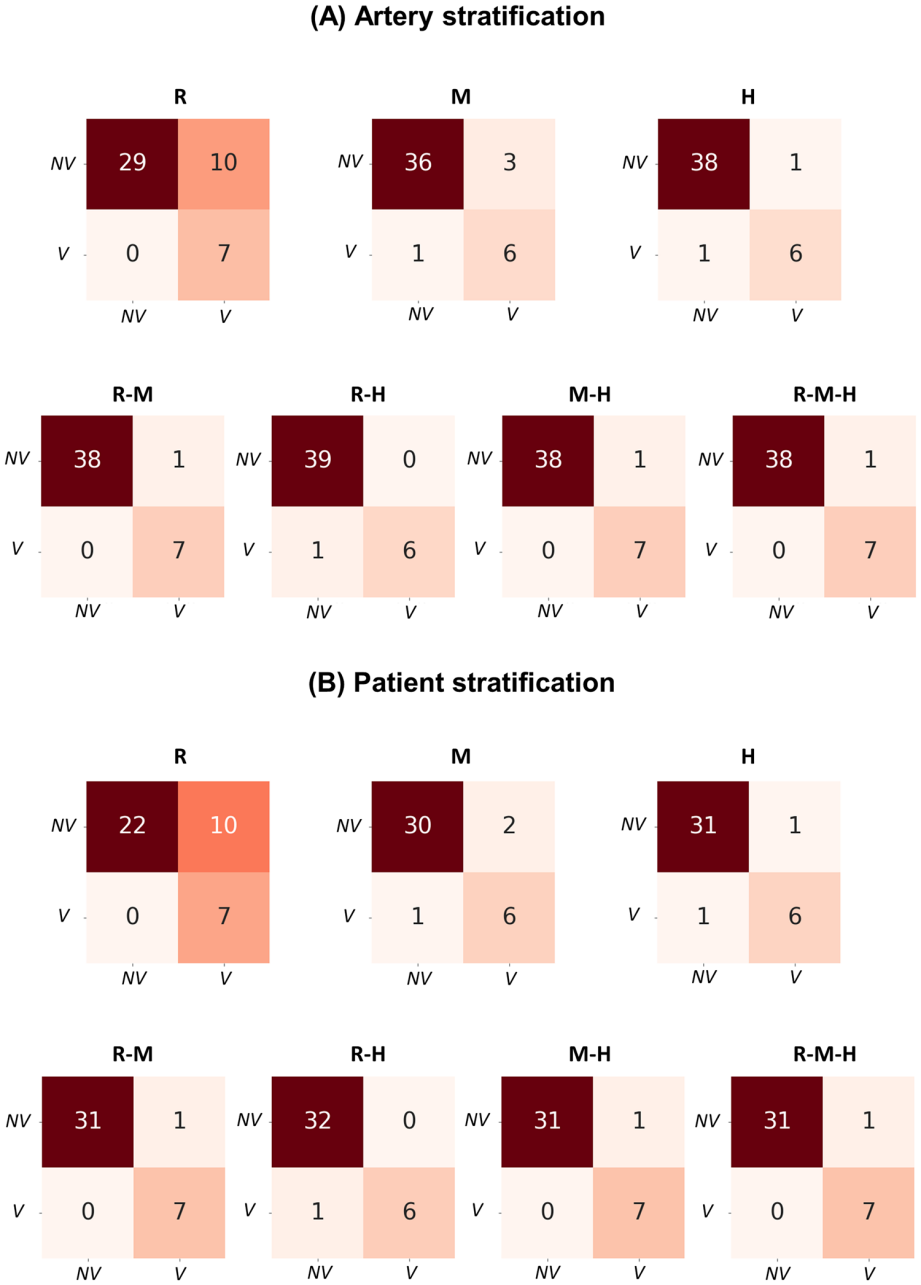
Confusion matrix of the radiomic (R), mechanical (M), hemodynamic (H), radiomic–mechanical (R–M), radiomic–hemodynamic (R–H), mechanical–hemodynamic (M–H), and radiomic–mechanical–hemodynamic (R–M–H) models for the artery stratification (**A**) and patient stratification (**B**) cases. For each confusion matrix, the true labels are on the row and the predicted labels on the column. NV: non-vulnerable; V: vulnerable

The KNN, DT, and ADA classifiers were selected for the radiomic, mechanical, and hemodynamic models, respectively. As regards the combined models, the KNN was selected for the radiomic–mechanical model, the ADA for the radiomic–hemodynamic model, the DT for the mechanical–hemodynamic, and for the radiomic–mechanical–hemodynamic models. Figure 7 presents the classification performance metrics for the radiomic, mechanical, hemodynamic, and combined models, for the artery and patient stratification tasks, and Fig. 8 shows the corresponding confusion matrices. The best performing individual model was the hemodynamic one with a balanced accuracy of 0.92, associated with the correct identification of 38 over 39 non-vulnerable arteries (31 over 32 low-risk patients) and 6 over 7 vulnerable arteries (6 over 7 high-risk patients). The mechanical model, with a balanced accuracy of 0.89, provided slightly lower performance on the non-vulnerable, low-risk cases (3 misclassified arteries and 2 misclassified patients), with equal performance on the vulnerable, high-risk cases (McNemar between the hemodynamic and mechanical model *p* > 0.05). Differently, the radiomic model was the only individual model correctly identifying all the vulnerable arteries and high-risk patients but presented a balanced accuracy of 0.87, due to the misclassification of 10 non-vulnerable, low-risk cases (*p*_R_H_ = 0.021, *p*_R_M_ > 0.05). Notably, the combination of the predicted probabilities from at least two individual models yielded an improvement in the performance metrics (with significance only compared to the radiomic model, *p*_RM_R_ = 0.012, *p*_RH_R_ = 0.012, *p*_MH_R_ = 0.004, *p*_RMH_R_ = 0.003). Indeed, in all the combined cases, only one artery and one patient were wrongly classified. With the exception of the radiomic-hemodynamic model, in all the combined models, the misclassified case was within the non-vulnerable, low-risk class, resulting in the optimization of the sensitivity and the overall performance (balanced accuracy of 0.99 for the artery stratification and 0.98 for the patient stratification).

## Discussion

The present study proposed the first combined radiomic–mechanic–hemodynamic approach for stratifying vulnerable coronary arteries and high-risk patients, by uniquely integrating radiomics, FEA, and CFD. The results obtained herein demonstrated the non-negligible role of hemodynamics, that, when considered alone, provided the best stratification performance, with only two misclassified cases and higher balanced accuracy with respect to the radiomic (+ 5.4% for the artery stratification, + 7.7% for the patient stratification) and mechanical (+ 3.2% for the artery stratification and + 1.1% for the patient stratification) models. When considering individual models, structural mechanics and hemodynamics alone outperformed radiomics, although this was the only individual model enabling the identification of all the vulnerable cases. Notably, combining the predictive probabilities of at least two models led to outstanding performance (balanced accuracy of 0.99), with only 1 case misclassified. However, combining the three models did not further improve performance compared to the radiomic–mechanical and mechanical–hemodynamic cases, where the mechanical model appears to leverage the key features of the individual radiomic and hemodynamic models.

In the literature, single radiomic, mechanical, and hemodynamic analyses were proposed to identify vulnerable coronary plaques or arteries. Several studies demonstrated the potential of radiomic features in stratifying vulnerable coronary plaques and identifying patients at risk of MACE, with performance similar to the ones reported here [38, 59]. Lin et al. [60] analyzed culprit and high-stenosis non-culprit plaques in patients with myocardial infarction and stable CAD, achieving an AUC = 0.84 on a test set of 35 plaques. Chen et al. [61] achieved an AUC = 0.95 in the identification of TCFA (labeled by OCT), considering a dataset of 43 plaques, and an AUC = 0.80 in the identification of vulnerable plaques, labeled by intravascular ultrasound, considering a dataset of 419 plaques [62]. Recently, Zheng et al. [63] reported an AUC = 0.87 in the detection of vulnerable plaques in 158 patients with stable angina pectoris.

Regarding structural mechanics analyses, our results are in line with previous findings highlighting the significant role of the plaque structural stress in stratifying coronary plaques and identifying patients at risk of MACE [22–28]. For instance, higher plaque structural stress was reported in non-calcified TCFA compared to thick-cap fibroatheromas [23], in ruptured compared to non-ruptured fibroatheromas [25, 30], in proximal rather than distal segments to the rupture site [25], and in plaques leading to MACE compared to non-MACE plaques [26]. Our recent study [22] also demonstrated that the peak plaque stress and dynamic pressure enabled robust detection of vulnerable coronary plaques, outperforming morphological features. Vulnerability associations with mechanical and morphological features were also confirmed by Guo et al. [31], in a single-patient analysis of 45 cross-sections. The recent findings of Gu et al. [34] highlighted that although enhancing the stratification of coronary plaques, the predictive accuracy of a model based on plaque structural stress alone was not sufficient, while the integration of different fluid dynamic and structural mechanics metrics significantly increased stratification performances [34]. Although in our analysis, structural mechanics features alone demonstrated high predictive performance, our results also reported an increase in the predictive power when hemodynamic features were integrated (particularly, TAWSS and OSI). As regards TAWSS, the area exposed to the lower 33rd percentiles, minimum, median, and mean values were the selected features, suggesting that medium-low TAWSS are relevant for stratification. Although WSS’ impact in plaque formation, progression, and stability has been largely investigated, its role remains controversial. In [33], local low WSS provided incremental risk stratification of untreated coronary lesions in high-risk patients with acute coronary syndrome, beyond measures of plaque burden, minimum lumen area, and morphology. However, higher WSS values were found in CCTA-derived high-risk rather than stable plaques [35]. In [36], WSS, axial plaque stress, pressure gradient across the lesion, and delta CT-derived fractional flow reserve (FFR-CT) across the lesion provided incremental and independent predictive ability of culprit from non-culprit lesions over CCTA-derived high-risk features and FFR-CT ≤ 0.80. Higher maximum WSS and WSS gradient were also found in plaques with erosion, compared with stable plaques [64].

Although previous correlations and statistical analyses highlighted the role of peak structural stress and WSS-based descriptors in coronary artery stratification, biomechanical-based ML models are lacking [17]. Despite the small dataset, we demonstrated not only that structural- and hemodynamic-based features can be effectively used for training ML classifiers with high stratification performance, but also that their integration, as well as the combination with radiomic variables, further improves the predictive power, highlighting the importance of synergistically integrating factors from different physics to characterize coronary artery vulnerability. Although being a preliminary investigation, this approach may pave the way toward a comprehensive multi-level analysis of CAD. In future, these findings should be validated on larger and multicentric datasets.

The study introduced substantial advancements compared to the previous investigation by the research group, focused on a radio-mechanical model [39]. First, the uncertainty related to the variability of the mechanical properties was addressed through a data-augmentation strategy, in which 100 material combinations were generated and considered as independent instances. Compared to the previous study in which the average mechanical properties were considered, an improvement was obtained, especially in the combined radiomic-mechanical model, with a balanced accuracy increase from 0.94 to 0.99 (5% increment) for the artery stratification and from 0.92 to 0.98 (6% increment) for the patient stratification. Second, the role of hemodynamics in characterizing vulnerable coronary arteries and high-risk patients was explored (while previously neglected), through the development of a ML model based on TAWSS, OSI, TransWSS, and RRT, computed via CFD.

The small, highly unbalanced dataset constitutes the main limitation of the present study, which prevents from drawing strong and robust conclusions. Indeed, only a leave-one-out cross-validation scheme was considered, limiting the validation of the results on a separate test set. Nevertheless, we can assume that the identified features are robust predictors of coronary artery vulnerability, being selected in more than 150 iterations over 161. Upon availability of additional data, a ML model can be trained considering the selected features and tested on the external dataset. Another limitation is the absence of longitudinal follow-up to determine whether the OCT-labeled vulnerable plaques subsequently ruptured or resulted in MACE. On one hand, OCT represents the gold standard for identifying culprit prone plaques, offering the unique advantage of high-resolution, plaque-level information that enables detailed characterization of vulnerable features. Thus, the CCTA-based models developed in this study, by predicting OCT-based characterization, provide a non-invasive and more accessible surrogate that could be applied at the population screening level. Nevertheless, incorporating MACE-based endpoints in future studies will be essential to determine the true prognostic value of the proposed framework for predicting clinical outcomes.

Once validated on larger cohorts and upon full automation, the proposed pipeline could be feasibly implemented in clinical practice for early identification of high-risk patients. Indeed, applying the framework to a new case would take approximately five hours, mainly due to the CFD analysis (~4 h), while segmentation, radiomic feature extraction, and FE analysis require about 20, 10, and 50 minutes, respectively, and final prediction of plaque vulnerability is instantaneous. To streamline the workflow, future analysis should be performed on identifying optimization targets for the CFD analysis to balance computational efficiency and accuracy, as done for the FE analysis. Indeed, simplified assumptions in the FE analysis (e.g., 2D rather than 3D and linear elastic isotropic rather than hyperelastic anisotropic material properties) ensured fast yet accurate simulations. In addition, the reference coronary geometry, used as the initial condition for applying diastolic or systolic pressure in the FE simulations, was reconstructed from CCTA images acquired when the vascular tissue was already under physiological load. As a result, it does not account for changes in vessel shape and position caused by the beating heart. Specifically, the reference geometry was loaded up to systolic pressure, and the biomechanical markers, i.e., the peak von Mises stress and maximum equivalent strain, were extracted at the end of both diastole and systole. To establish a zero-pressure condition for computational simulations, an inverse elastostatics methodology could be employed to estimate the unloaded geometry. This approach would allow for a more accurate assessment of biomechanical risk.

Our model, by predicting OCT-defined plaque vulnerability, extends beyond functional assessment, providing complementary information to FFR measurements and potentially identifying high-risk plaques that would otherwise remain undetected. Indeed, although low FFR values and high pullback pressure gradients have recently been associated with OCT-defined vulnerability in functionally significant lesions (FFR < 0.80) [65], vulnerable plaques may also be non-stenotic and exhibit outward remodeling, thereby escaping detection by FFR-based assessment. Finally, although not yet implemented, the workflow automation is technically straightforward, and the adopted segmentation software is already in clinical use, thus facilitating the workflow translation to clinical practice.

In conclusion, the present study proposed the first ML approach, integrating radiomics, structural mechanics, and hemodynamics for the detection of coronary vulnerable arteries and high-risk patients. Although the analysis was based on only 39 patients, the results suggest the potentiality of the approach and pave the way toward a synergistic multi-level analysis of coronary plaques. The main findings of the investigation include the following: (i) the superior overall performance of hemodynamics alone, compared to structural mechanics and radiomics; (ii) the unique capability of radiomics alone in detecting all the vulnerable cases, despite the lower performance in classifying the non-vulnerable cases, and (iii) the improved predictive power obtained with the integration of at least two over three variable types. Future efforts should focus on validating the promising initial results.

## Supplementary Information

Below is the link to the electronic supplementary material.

Supplementary file1 (PDF 703 kb)

## Data Availability

All relevant data and materials have been included in the article. Further inquiries can be directed to the corresponding authors.

## Declarations

### Clinical Trial

The present study does not involve a clinical trial.
