# Supplementary material for: Advancing Coronary Risk Assessment Through Combined Radiomic, Mechanical, and Hemodynamic Analysis
Source: Ann Biomed Eng. 2026 Feb 23;54(7):2288–304. doi: 10.1007/s10439-026-03994-1 (PMC13290838; doi:10.1007/s10439-026-03994-1)
Supplement: Supplementary file 1 — Supplementary file1 (PDF 703 kb) [file 10439_2026_3994_MOESM1_ESM.pdf]

Advancing Coronary Risk Assessment Through Combined Radiomic, Mechanical, and Hemodynamic Analysis

- Supplementary Material –

Anna Corti[1,*], Marco Stefanati[2], Vittorio Lissoni[2], Matteo Leccardi[1], Francesco Bruno[3], Alessandro Depaoli[4], Pietro Cerveri[1,5], Francesco Migliavacca[2], Valentina D.A. Corino[1,6], José F. Rodriguez Matas[2], Luca Mainardi[1], Gabriele Dubini[2]

1. Department of Electronics, Information and Bioengineering, Politecnico di Milano, Milan, Italy
2. Laboratory of Biological Structure Mechanics (LaBS), Department of Chemistry, Materials and Chemical Engineering "Giulio Natta", Politecnico di Milano, Milan, Italy
3. Division of Cardiology, Department of Medical Sciences, "Città della Salute e della Scienza di Torino" Hospital, University of Turin, Turin, Italy
4. Radiology Unit, Department of Surgical Sciences, "Città della Salute e della Scienza di Torino" Hospital, University of Turin, Turin, Italy
5. Department of Industrial and Information Engineering, University of Pavia, Pavia, Italy
6. Cardiotech Lab, Centro Cardiologico Monzino IRCCS, Milan, Italy

***Address for correspondence:**

Anna Corti, PhD

Department of Electronics, Information and Bioengineering, Politecnico di Milano, Via Ponzio 34/5, 20133 Milan, Italy

anna.corti@polimi.it

Patient data acquisition

CCTA was performed using a Revolution CT (GE Healthcare, Milwaukee, WI, USA) with a 256 detector-row scanner with a spatial resolution of 0.23 mm and the ability to acquire 512 slices (117.8 mm slice interval) in 0.28s. OCT imaging was performed with a CT system (LightLab Imaging Inc/St Jude Medical, Westford, MA, USA) connected to a catheter C7 Dragonfly with motorized catheter pull back (20 mm/s) after the administration of intracoronary glycerin trinitrate. OCT images were analyzed by an expert operator at the time of the procedure, and post-hoc offline by an independent investigator who was blinded to the clinical presentation.

Structural mechanics analysis

For the convergence of structural mechanics analysis simulations, the sensitivity analysis is reported in Table S1. The simulations were performed with an average element size of 0.0131 mm (maximum of 0.0502 mm and minimum of 0.00125 mm) and the maximum distance between the cross-sections was of 7.57 mm and the minimum of 0.32 mm. The simulations were carried out on 8 Intel Xeon64 CPUs and 16 GB of RAM. For a single simulation approximately 30 seconds (27.38 ± 10.35 s) were required to compute the diastolic-systolic cycle.

	**Mesh size (mm)**	**Total number of elements**	**max_$pMISES^{dyn}$ (kPa)**	**% diff.**	**max_ε_{eqv}^{dyn} (-)**	**% diff.**
Mesh 1	2.29E-02	40748	236.4295	8.55	0.1137	4.92
Mesh 2	1.42E-02	110220	219.1356	0.61	0.1164	2.67
Mesh 3	7.56E-03	386902	217.8116		0.1196	

Table S1. Percentage differences in the max_$pMISES^{dyn}$ and max_ε_{eqv}^{dyn} for consecutive refinements of the LAD (left anterior descending) coronary mesh.

Once the finite element (FE) model is established, an elastic analysis is conducted to extract structural biomarkers across the arterial cross-sections. The coronary region is defined as a domain $\Omega \subset \mathbb{R}^2$ with boundary $\partial\Omega$ subdivided into multiple subdomains Ω_1, Ω_2, …, representing distinct

tissue types, such as calcium, fibrous-fatty plaque, fibrous plaque, necrotic core, and healthy arterial wall. Assuming no body forces, the mechanical equilibrium is governed by:

$$\nabla \cdot \sigma(\boldsymbol{u}) = 0 \qquad \text{in } \Omega, \tag{1}$$

with boundary conditions:

$$\sigma(\boldsymbol{u}) \cdot \hat{\boldsymbol{n}} = \boldsymbol{t} \qquad \text{on } \Gamma_N, \tag{2}$$

$$\sigma(\boldsymbol{u}) \cdot \hat{\boldsymbol{n}} = -\alpha\boldsymbol{u} \qquad (\alpha > 0) \qquad \text{on } \Gamma_R, \tag{3}$$

where $\boldsymbol{\sigma}$ is the Cauchy stress tensor and $\boldsymbol{u}$ is the displacement field; $\boldsymbol{t}$ is the surface traction, α is the elastic bed coefficient, and $\hat{\boldsymbol{n}}$ is the outward unit normal to the boundary. Equation (2) defines the Neumann boundary condition, applied at the lumen surface to simulate patient-specific blood pressure, either diastolic or systolic. In contrast, equation (3) introduces a Robin boundary condition, which models the mechanical interaction between the artery and surrounding tissue through an elastic foundation. To enable realistic vessel expansion and eliminate rigid body modes from the finite element system, the elastic bed coefficient α was set to 0.01 kPa/mm, approximately 0.01% of the medial layer's Young modulus (Gahima et al., 2023 [1]). Together, the Neumann and Robin conditions span the entire boundary of the domain, such that $\partial\Omega = \Gamma_N \cup \Gamma_R$.

Plaque structural stress (PSS) is quantified using the peak von Mises stress (pMISES), defined as the maximum value across all elements:

$$pMISES = \max_{i=1,N_e} MISES_i^t, \tag{4}$$

where the subscript i is the element number, Ne the total number of elements in the model, and $t \in \{dia, sys, dyn\}$ denotes the time frame (diastolic, systolic, or dynamic). Dynamic stress is computed as:

$$pMISES^{dyn} = \max_{i=1,N_e}\left(MISES_i^{sys} - MISES_i^{dia}\right), \tag{5}$$

with dynamic pressure defined as $P_{dyn} = P_{sys} - P_{dia}$ (mean ± standard deviation of 62 ± 18 mmHg).

Similarly, plaque equivalent strain (PES) is assessed via the maximum equivalent strain across elements. Both von Mises stress and equivalent strain are computed at the centroid of each triangular element using:

$$MISES = \frac{1}{\sqrt{2}}\sqrt{\left(\sigma_{xx} - \sigma_{yy}\right)^2 + \left(\sigma_{yy} - \sigma_{zz}\right)^2 + (\sigma_{zz} - \sigma_{xx})^2 + 6\left(\sigma_{xy}^2 + \sigma_{yz}^2 + \sigma_{zx}^2\right)}, \quad (6)$$

$$\varepsilon_{eqv} = \frac{1}{\sqrt{2}(1+\nu)}\sqrt{\left(\varepsilon_{xx} - \varepsilon_{yy}\right)^2 + \left(\varepsilon_{yy} - \varepsilon_{zz}\right)^2 + (\varepsilon_{zz} - \varepsilon_{xx})^2 + 6\left(\varepsilon_{xy}^2 + \varepsilon_{yz}^2 + \varepsilon_{zx}^2\right)}, \quad (7)$$

where ν is Poisson's ratio (i.e., the negative ratio of circumferential strain to longitudinal strain) of the corresponding plaque tissue.

To avoid artifacts caused by abrupt stiffness transitions, elements composed of calcium or adjacent to calcified regions are excluded from the computation of peak stress and strain. This ensures more reliable assessment by minimizing artificial stress concentrations near highly rigid zones.

Computational fluid dynamics analysis

A sensitivity analysis for the CFD simulations was performed on the left anterior descending branch for one representative case (Figure SA). A simplified setup was adopted, applying the average left coronary flow as the inlet boundary condition and a zero-pressure condition (0 mmHg) at the outlet. The inlet blood pressure and outlet flow obtained with progressively finer mesh sizes were extracted and compared.

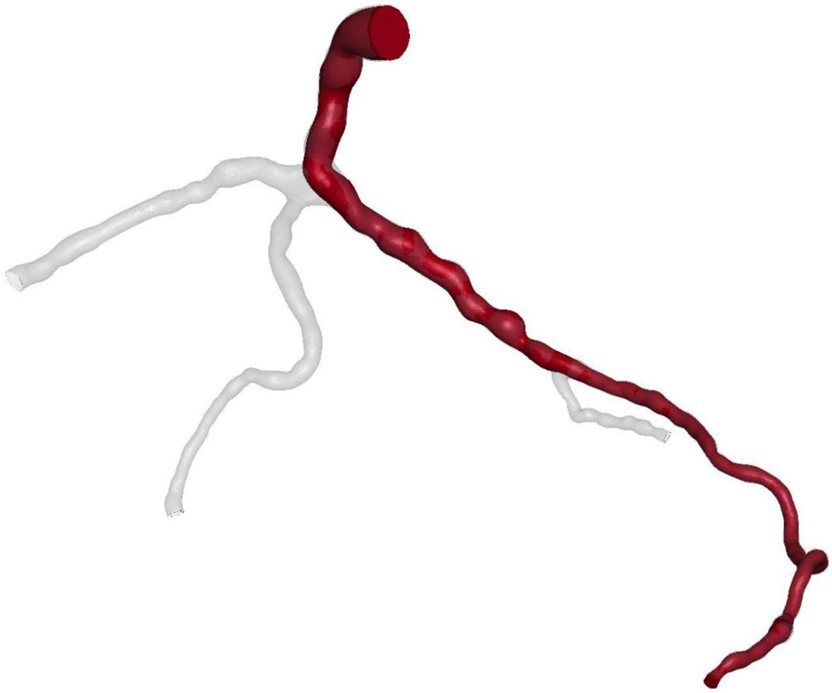

Figure S1. Coronary branch investigated for the sensitivity analysis with the whole coronary tree in transparency

For the convergence of CFD simulations, the sensitivity analysis is reported in Table S2 and S3.

	**Mesh size (mm)**	**Total number of elements**	**Inlet Pressure (MPa)**	**% difference**
Mesh 1	0.7	82919	0.01000	
Mesh 2	0.6	136093	0.00656	34.4
Mesh 3	0.5	176801	0.00675	-2.9
Mesh 4	0.4	314707	0.00680	-0.7

Table S2. Percentage differences in the inlet pressure for consecutive refinements of the blood shell mesh.

	**Mesh size (mm)**	**Total number of elements**	**Outlet flow (cc/s)**	**% difference**
Mesh 1	0.7	82919	1.991	
Mesh 2	0.6	136093	1.880	5.84
Mesh 3	0.5	176801	1.877	0.16
Mesh 4	0.4	314707	1.869	0.43

Table S3. Percentage difference in the outlet flow for consecutive refinements of the blood shell mesh.

The CFD simulations were performed with an average element size of 0.45 mm, and the resulting model solved using a fractional step time discretization and linear discretization for both pressure and

velocity and an implicit scheme with a constant timestep of 0.05 ms. For each timestep, convergence was achieved when all residuals were below 10^{-10}. CFD simulations were carried out on 28 Intel Xeon64 CPUs and 250 GB of RAM, using the commercial finite element solver LS-DYNA 971 Release 14.0 (ANSYS, Canonsburg, PA, USA). For each anatomy approximately 4 hours were required to compute the three simulated cardiac cycles.

CFD simulations workflow

The following section briefly describes the workflow used for CFD simulation setup and results extraction, illustrated through two exemplifying coronary arteries, displayed in Figure S2.

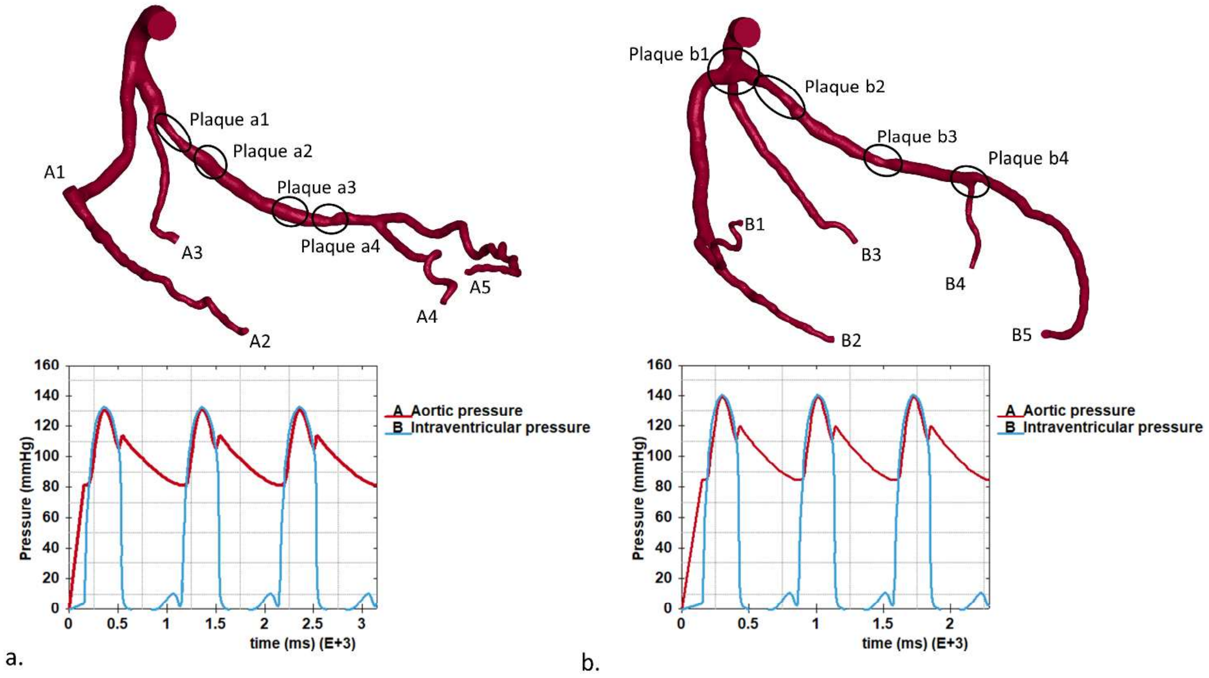

Figure S2. Two examples of investigated coronaries with their corresponding aortic and intraventricular pressures and details of plaque components position.

After generating the surface mesh with a target element size of 0.45 mm, the diameters of each coronary branch are measured near the bifurcations. For each case, the theoretical aortic pressure waveform is scaled according to the patient-specific systolic and diastolic pressures and heart rate. The resulting curve is applied as the inlet boundary condition and used to compute the average pressure. The average flow rate is derived from literature values of mean coronary flow. The total coronary resistance is then calculated as:

$$Rtot = \frac{Paverage(patient - specific)}{Qaverage(literature)}$$

The total resistance is split at the difference outlet following the same methodology presented in Taylor et al. [2]. The resistance at each outlet is determined according to the following morphometric law, which states that resistance is inversely proportional to the cube of the vessel diameter:

$$R_i = \left(\frac{Diameter_i^{\ 3}}{\sum Diameter^3}\right)^{-1} * Rtot$$

The compliance of lumped parameter models at each outlet are calculated with the following formula:$Ci = \frac{100}{Ri*dt}$

While resistances are necessary to simulate the right average pressure and flow values, compliance affects the smoothness and amplitude of the resulting curves. In our previous work [3] we found that compliances estimated with this formula guaranteed the best match with theoretical coronary flow curves. Five-element lumped parameter models are completed using intraventricular pressure curves. For each patient, this curve is obtained by scaling a theoretical intraventricular pressure waveform from the literature according to the patient-specific systolic pressure. The resulting curves for the two example cases are shown in Figure S1. The total resistance and compliance values at each outlet are then distributed into specific R1, R2, R3, C1, and C2 components according to percentage ratios reported in the literature [4]. The measured diameters, estimated resistances and compliances at each outlet for the two examples are reported in table S4.

	outlet	Diameters (mm)	R1 (10^{-3} MPa ms/mm^3)	R2 (10^{-3} MPa ms/mm^3)	R3 (10^{-3} MPa ms/mm^3)	C1 (10^3 mm^3/MPa)	C2 (10^3 mm^3/MPa)
Coronary A	A1	2.89	9.57	15.6	4.78	7.35	59.4
	A2	2.35	17.9	29.0	8.94	3.93	31.8
	A3	1.70	47.0	76.4	23.5	1.50	12.1
	A4	1.80	39.9	64.5	20.0	1.76	14.3
	A5	1.87	30.0	48.8	15.0	2.34	19.0
Coronary B	B1	2.70	15.6	25.3	7.79	4.52	36.6
	B2	2.70	15.6	25.3	7.79	4.52	36.6
	B3	2.60	17.4	28.4	8.72	4.03	32.6

	B4	1.41	109	178	54.7	0.643	5.21
	B5	2.40	22.1	36.0	11.1	3.17	25.7

Table S4. Diameters measured for each coronary branch, resistances and compliances for each lumped parameter model of the outlet sections shown in figure S1.

Simulation results were exported in VTK format, compatible with ParaView. Velocity gradients on the vessel walls were extracted for each element and stored in text files at every output time using a ParaView-compatible Python script. Values only from the third simulated cardiac cycle were extracted. A second Python code was then used to compute wall shear stress values for each element at each time step. These data were subsequently processed to calculate TAWSS, OSI, RRT, and TransWSS for all wall elements. Once results were obtained for all cases, the 33rd and 66th percentile values of each biomarker were extracted from the full set of wall element results. A MATLAB script was then employed to isolate vessel wall segments corresponding to plaques, whose locations had been previously identified by an expert radiologist. For each plaque region, the minimum, maximum, average, standard deviation and median values were computed, together with the surface area exposed to values below the 33rd percentile and above the 66th percentile. These results are reported in the following tables for the plaque elements of the examples.

	Plaque a1	Plaque a2	Plaque a3	Plaque a4	Plaque b1	Plaque b2	Plaque b3	Plaque b4
Average (Pa)	5.75E-01	2.95E-01	2.56E-01	1.11E+00	3.61E-01	5.47E-01	3.36E-01	5.09E-01
Median (Pa)	5.07E-01	2.94E-01	2.70E-01	1.35E+00	3.26E-01	5.09E-01	3.46E-01	4.90E-01
standard deviation (Pa)	3.29E-01	4.77E-02	9.11E-02	5.32E-01	1.31E-01	2.88E-01	1.83E-01	2.88E-01
Minimum (Pa)	7.52E-02	1.80E-01	7.20E-02	1.15E-01	1.16E-01	6.04E-02	2.44E-02	4.62E-02
Maximum (Pa)	1.61E+00	4.33E-01	5.25E-01	1.91E+00	8.02E-01	1.16E+00	8.13E-01	1.34E+00
area<percentile33 (mm2)	2.01E+01	6.53E+01	7.25E+01	1.16E+01	8.29E+01	2.98E+01	9.44E+01	4.32E+01
area>percentile66 (mm2)	1.34E+01	0.00E+00	0.00E+00	3.46E+01	0.00E+00	1.81E+01	0.00E+00	1.73E+01
area<percentile33 %	2.92E-01	9.27E-01	8.97E-01	1.98E-01	7.31E-01	3.51E-01	5.30E-01	3.66E-01
area>percentile66 %	1.95E-01	0.00E+00	0.00E+00	5.92E-01	0.00E+00	2.13E-01	0.00E+00	1.47E-01

Table S5. TAWSS results extracted for each plaque in the two examples

	Plaque a1	Plaque a2	Plaque a3	Plaque a4	Plaque b1	Plaque b2	Plaque b3	Plaque b4
average	1.79E-01	1.72E-01	1.81E-01	1.73E-01	5.90E-02	5.85E-02	6.36E-02	8.83E-02
median	1.72E-01	1.72E-01	1.76E-01	1.72E-01	5.66E-02	5.72E-02	5.76E-02	6.71E-02
standard deviation	3.33E-02	9.56E-04	1.57E-02	6.89E-03	9.85E-03	1.08E-02	3.22E-02	5.86E-02
minimum	1.60E-01	1.69E-01	1.63E-01	1.63E-01	5.39E-02	5.15E-02	4.77E-02	5.07E-02
maximum	4.14E-01	1.77E-01	2.67E-01	2.34E-01	1.77E-01	2.25E-01	4.03E-01	4.50E-01

area<percentile33 (mm2)	0.00E+00	0.00E+00	0.00E+00	0.00E+00	0.00E+00	0.00E+00	0.00E+00	0.00E+00
area>percentile66 (mm2)	6.89E+01	7.04E+01	8.09E+01	5.85E+01	1.05E+00	1.23E+00	7.31E+00	2.01E+01
area<percentile33 %	0.00E+00	0.00E+00	0.00E+00	0.00E+00	0.00E+00	0.00E+00	0.00E+00	0.00E+00
area>percentile66 %	1.00E+00	1.00E+00	1.00E+00	1.00E+00	9.25E-03	1.45E-02	4.10E-02	1.70E-01

Table S6. OSI results extracted for each plaque in the two examples

	Plaque a1	Plaque a2	Plaque a3	Plaque a4	Plaque b1	Plaque b2	Plaque b3	Plaque b4
Average (Pa)	3.43E-03	6.93E-04	2.66E-03	5.26E-03	2.38E-03	2.81E-03	1.99E-03	8.36E-03
Median (Pa)	1.44E-03	4.74E-04	1.78E-03	3.59E-03	1.05E-03	1.51E-03	1.29E-03	5.34E-03
standard deviation (Pa)	5.66E-03	6.93E-04	2.58E-03	5.53E-03	3.94E-03	3.60E-03	2.06E-03	9.10E-03
Minimum (Pa)	2.00E-06	1.00E-06	2.00E-06	2.00E-06	1.00E-06	4.00E-06	1.00E-06	1.00E-06
Maximum (Pa)	4.67E-02	4.59E-03	1.76E-02	3.73E-02	3.65E-02	3.70E-02	1.55E-02	8.14E-02
area<percentile33 (mm2)	2.82E+01	5.37E+01	2.69E+01	8.89E+00	5.79E+01	3.25E+01	7.63E+01	1.43E+01
area>percentile66 (mm2)	1.25E+01	0.00E+00	1.43E+01	2.05E+01	1.24E+01	1.42E+01	1.55E+01	5.97E+01
area<percentile33 %	4.10E-01	7.63E-01	3.32E-01	1.52E-01	5.10E-01	3.83E-01	4.28E-01	1.21E-01
area>percentile66 %	1.82E-01	0.00E+00	1.76E-01	3.51E-01	1.10E-01	1.68E-01	8.68E-02	5.06E-01

Table S7. TransWSS results extracted for each plaque in the two examples

	Plaque a1	Plaque a2	Plaque a3	Plaque a4	Plaque b1	Plaque b2	Plaque b3	Plaque b4
Average (s/Pa)	4.70E+00	5.32E+00	7.45E+00	2.27E+00	3.54E+00	3.04E+00	6.56E+00	5.83E+00
Median (s/Pa)	3.02E+00	5.19E+00	5.71E+00	1.12E+00	3.46E+00	2.21E+00	3.26E+00	2.35E+00
standard deviation (s/Pa)	7.76E+00	9.13E-01	4.14E+00	2.40E+00	1.27E+00	2.54E+00	1.25E+01	1.46E+01
Minimum (s/Pa)	9.45E-01	3.52E+00	2.90E+00	7.96E-01	1.41E+00	9.74E-01	1.39E+00	8.48E-01
Maximum (s/Pa)	7.34E+01	8.51E+00	2.47E+01	1.62E+01	1.34E+01	3.01E+01	1.89E+02	2.10E+02
area<percentile33 (mm2)	9.66E+00	0.00E+00	0.00E+00	3.18E+01	4.17E+00	2.95E+01	6.82E+00	2.88E+01
area>percentile66 (mm2)	2.01E+01	6.53E+01	7.28E+01	1.17E+01	2.24E+01	1.86E+01	7.30E+01	3.22E+01
area<percentile33 %	1.40E-01	0.00E+00	0.00E+00	5.43E-01	3.68E-02	3.47E-01	3.83E-02	2.44E-01
area>percentile66 %	2.92E-01	9.27E-01	9.01E-01	2.00E-01	1.98E-01	2.19E-01	4.10E-01	2.73E-01

Table S8. RRT results extracted for each plaque in the two examples

References

[1] Gahima S, Díez P, Stefanati M, Rodríguez Matas JF, García-González A, An Unfitted Method with

Elastic Bed Boundary Conditions for the Analysis of Heterogeneous Arterial Sections. Mathematics. 2023; 11:. https://doi.org/10.3390/math11071748.

[2] Taylor CA, Fonte TA, Min JK, Computational fluid dynamics applied to cardiac computed tomography for noninvasive quantification of fractional flow reserve: scientific basis. J. Am. Coll. Cardiol. 2013; 61: 2233–2241. https://doi.org/10.1016/j.jacc.2012.11.083.

[3] Lissoni V, Luraghi G, Stefanati M, Rodriguez Matas JF, Migliavacca F, Computational methods used to investigate atherosclerosis progression in coronary arteries: structural FEA, CFD or FSI. Comput. Methods Programs Biomed. 2025; 270: 108959. https://doi.org/10.1016/j.cmpb.2025.108959.

[4] Sankaran S, Esmaily Moghadam M, Kahn AM, Tseng EE, Guccione JM, Marsden AL, Patient-specific multiscale modeling of blood flow for coronary artery bypass graft surgery. Ann. Biomed. Eng. 2012; 40: 2228–2242. https://doi.org/10.1007/s10439-012-0579-3.
